# Longitudinal structure-function analysis of molecularly-confirmed *CYP4V2* Bietti Crystalline Dystrophy

**DOI:** 10.1038/s41433-023-02791-7

**Published:** 2023-10-28

**Authors:** Riccardo Cheloni, Neil Clough, Daniel Jackson, Mariya Moosajee

**Affiliations:** 1 UCL Institute of Ophthalmology, London, EC1V 9EL UK; 2 Moorfields Eye Hospital NHS Foundation Trust, London, EC1V 2PD UK; 3 The Francis Crick Institute, London, NW1 1AT UK

**Keywords:** Hereditary eye disease, Prognostic markers, Outcomes research

## Abstract

**Objectives:**

Bietti Crystalline Dystrophy (BCD) is an autosomal recessive progressive retinal disease caused by mutations in *CYP4V2*. We have characterised the natural history including structural and functional measures to identify potential outcome metrics for future clinical trials.

**Methods:**

Molecularly-confirmed BCD patients with biallelic variants in *CYP4V2* were retrospectively identified from Moorfields Eye Hospital (UK). Clinical details including results of molecular genetic testing, best-corrected visual acuity (BCVA) and spectral-domain optical coherence tomography (OCT) scans were extracted. From OCT scans, ellipsoid zone (EZ) measures, foveal thickness of the whole retina, outer retina and choroid were measured. Age-related changes of clinical parameters were assessed with linear mixed models.

**Results:**

Twenty-eight BCD patients were identified, with median age at baseline of 37 years (interquartile range [IQR]: 30–49.5). Median follow-up was 7.7 years (IQR: 3.4–14.5). Most patients (41.7%) showed chorioretinal atrophy at baseline. All OCT parameters showed significant age-related loss (*p* < 0.05), with EZ measures and choroidal thickness displaying the most rapid degeneration (2.3–3.3% per year vs 0.6–1.5% per year). Median BCVA was 0.2 LogMAR (IQR: 0–0.5) at baseline and showed small age-related loss ( + 0.016 LogMAR per year, *p* = 0.0019). Patients exhibited substantial phenotypic variability.

**Conclusions:**

BCD presents between age 25 and 40, and slowly progresses to an advanced chorioretinal atrophy and vision loss by age 60. BCVA may be preserved until late, and is seemingly poorly representative of disease progression. OCT parameters capturing EZ and choroid changes may afford more suitable trial outcome measures.

## Introduction

Bietti Crystalline Dystrophy (BCD) is a progressive inherited retinal disorder first described in 1937 [1]. Although the prevalence of BCD may be underestimated, it varies with ethnicity from around 1 in 4,500,000 subjects in Europe to 1 in 67,000 in China [2, 3]. BCD exhibits autosomal recessive inheritance, and variants in the *CYP4V2* gene (OMIM *608614) are known to be causative [4, 5]. *CYP4V2* encodes for a 525 amino-acid protein that is a member of the cytochrome p450 group of enzymes involved in fatty acid and steroid metabolism [6, 7]. Immunohistochemistry analyses suggest CYP4V2 is highly expressed in the choroid and retinal pigment epithelium (RPE), with relatively lower expression levels in the neural retina and corneal epithelial cells [7]. Accordingly, RPE cells are considered the major target of BCD [2], and dysfunctional lipid metabolism is considered the main disease mechanism [6, 7].

White-yellow retinal, and occasionally corneal, crystals are the hall-mark sign of BCD, which is otherwise characterised by RPE degeneration, thinning of choroidal vessels, and outer retinal degeneration [8, 9]. Disease onset spans the 2nd–4th decade of life [2, 3], with reduced vision, night-blindness, visual field loss and impaired colour vision reported at presentation [8–12]. There is a qualitative understanding of BCD natural progression [8, 9, 13–15], with retinal crystal formation representing an early product of RPE dysfunction. Crystals are mainly found in the posterior pole within the RPE-Bruch’s membrane complex at early stages [8, 9, 15]. With disease progression crystals are superseded by RPE atrophy, seen as hypo-fluorescence on fundus autofluorescence (FAF) or RPE and outer retinal atrophy in spectral-domain optical coherence tomography (SD-OCT) scans [8]. RPE atrophy is followed by atrophy of choroidal vessels [14, 16].

BCD is currently untreatable and progresses to legal blindness by the 5th or 6th decade of life [8–12]. However, a few therapeutic avenues have proved promising in BCD’s disease models, either via metabolic-lipid-modifying routes or gene therapy [2, 17–20]. Although key clinical signs and the qualitative progression pattern of BCD are known [8, 9, 13–15], there is a paucity of longitudinal studies assessing quantitative measures of disease progression [21, 22]. Herein, we characterised the natural history of patients with molecularly-confirmed BCD to provide better prognostication for patients and insights into outcome measures for future clinical trials.

## Methods

### Setting and study population

Patients with disease-causing variants in *CYP4V2* were identified from the Inherited Eye Disease Database at Moorfields Eye Hospital NHS Foundation Trust, London, UK. All participants provided informed consent (12/LO/0141) and all procedures adhered to the tenets of the Declaration of Helsinki. Twenty of the 28 patients included in this study were also reported in an older observational study by our institution, and corresponding ID numbers are reported for cross-reference [8].

Data for this retrospective study was collected as part of standard care and accessed through electronic and written medical records. All participants underwent a full eye examination at each visit, including best-corrected visual acuity (BCVA) and retinal imaging. Molecular confirmation of *CYP4V2* variants resulting in a BCD diagnosis was the only inclusion criteria.

### Retinal imaging

Spectralis (Heidelberg Engineering, Heidelberg, Germany) was used to obtain macular SD-OCT scans (19 B-scans, 512 A-scans/B-scans; 97 B-scans, 1024 A-scans/B-scans) and FAF imaging. Built-in automated retinal tracking was used to reduce measurement noise. Wide-field pseudo-colour fundus photos were collected with Optos California (Optos plc, Dunfermline, UK).

OCT scans were only used if gradable, with fovea centration and free from artefacts (e.g. poor optical quality, ocular movements/blinks). Outer retinal layers were qualitatively assessed as previously described [9] including: i) ellipsoid zone (EZ) and RPE continuous/intact; ii) localized EZ disruption; iii) focal residual EZ and/or RPE; iv) severe atrophy. Retinal comorbidities such as choroidal neovascularisation, epi-retinal membranes and cystoid macular oedema (CMO) were also recorded.

Quantitative OCT metrics were extracted by a senior grader considering the horizontal foveal B-scan (Supplementary Fig. 1). EZ parameters were manually measured using custom programs written in MATLAB (Version 9.6.0, The MathWorks Inc., Natick, MA). EZ width was the sum of nasal and temporal widths, identified as the distance between the first EZ interruption on either side of the fovea, where EZ and RPE could not be differentiated. To account for focal EZ loss, we also measured the proportion-preserved EZ. This parameter ranges between 0-100%, and is obtained by dividing the cumulative sum width of residual EZ layer in the foveal B-scan by the whole B-scan width. As proposed elsewhere [23], we regarded only clear EZ loss as interruption, and signal attenuation was not considered a break. Retinal and choroidal thicknesses were measured manually using Spectralis’ inbuilt measuring tool. For greater consistency we considered thickness as the vertical distance between retinal layers of interest at the fovea [24, 25]. Central retinal thickness (CRT) was measured between the inner limiting membrane and the RPE. Thickness of photoreceptor and RPE complex (PR + RPE) was taken between the external limiting membrane and the RPE [25, 26]. Lastly, in patients with detectable choroid-scleral junction, choroidal thickness was measured between the posterior RPE border and anterior border of the sclera, visible as the outermost dark to bright transition from tubular choroidal texture to uniform scleral texture [27–29].

Disease severity in fundus photo was graded qualitatively at baseline according to the Yuzawa classification system (Supplementary Table 1) [30]. We attempted to objectively quantify retinal crystals in fundus photos (Supplementary File 1). However, most participants presented with severe chorioretinal atrophy at baseline, leaving the sample size too small for further quantitative analysis (*n* = 4). FAF images were also graded qualitatively as proposed before [8, 9]: i) normal autofluorescence; ii) hyper- or hypo-autofluorescence within posterior pole (within arcades); iii) hypo-autofluorescence beyond posterior pole; iv) severe autofluorescence loss.

### Statistical analysis

All statistical analyses were performed in *R* [31], and statistical significance was considered when *p* < 0.05. Most data series did not follow a normal distribution, therefore non-parametric statistics were used throughout.

BCVAs were converted to LogMAR, and values of counting fingers, hand movement, light-perception, and no light-perception, were converted to 2.6, 2.7, 2.8 and 2.9 LogMAR, respectively [26, 32]. Inter-ocular relationship of quantitative measures was assessed with Spearman’s correlation (*ρ*) at baseline, last visit and for changes from baseline. Only measurements from right eyes were considered for further analyses.

We performed simple linear regression to assess age-related changes from cross-sectional examinations at baseline. We also conducted a longitudinal analysis with only data from patients with 2 visits or more using linear mixed models to account for repeated measures, using the *nlme* R package [33]. We built individual models for each quantitative measure, using the parameter of interest (e.g., EZ width) as outcome measure, age at visit as fixed effect and patient ID as random effect (intercept and slope). BCVA scores ≤2.6 were excluded from this analysis due to their limited reliability [34]. EZ progression analysis was limited to participants showing measurable EZ parameters at baseline.

Structure-function and structure-structure relationships at baseline were tested with Spearman’s correlation, and family-wise Bonferroni correction was applied to control for multiple testing. We explored genotype-phenotype correlation by testing differences in quantitative measures between genetic status. Differences were assessed with linear mixed models using data at all visits and controlling for age. A basic model was built with age as fixed effect and patient ID as random effect (intercept). A second model was then built adding genetic status as fixed effect and we tested significance of differences by genetic status with likelihood ratio tests. Lastly, we estimated time to exhaustion of BCVA with Kaplan-Meier analysis using the *Survival* R package [35], and considering an outcome of sight impairment (BCVA ≥ 1.0 LogMAR). We applied left and right-censoring and age at the time of the visit was used as time variable.

## Results

We included 28 patients from 23 unrelated families. Demographic and clinical characteristics are summarised in Supplementary Table 1. There were 16 (57.1%) females, and the majority of patients were of South Asian ethnicity (*n* = 15, 62.3%). First symptoms were reported at median age of 29 years (interquartile range (IQR): 25–38), most commonly being nyctalopia and peripheral field loss (56.0% and 28.0%, respectively). Corneal crystals were present in 33% of patients (*n* = 9).

### Molecular characteristics

Fifteen variants were identified (Table 1, Supplementary Fig. 2). Four variants were novel: 2 splice site (c.414-1 G > A, c.985+3 A > G), 1 nonsense (c.279 G > A, [p.Trp93*]) and 1 deletion (c.637_641del, [p.Ser213*]). The most frequent were c.197 T > G (p.Met66Arg) and c.802-8_810delinsGC, affecting 15 patients in 10 unrelated families and 5 patients in 5 unrelated families, respectively.

**Table 1:** Details of individual patients and *CYP4V2* genetic variants identified in the study population (based on NM_207352.4).

ID	Family ID	Gender	Ethnicity	Variant and predicted protein change	Variant type	BCVA at (age)
01	19152	M	White other	c.76 G > C, p.(Gly26Arg)	Missense	0.1 (33 y)
				c.802-8_810delinsGC, del of exon7	Deletion	
02	15147	F	White other	c.77 G > A, p.G(ly26Asp)	Missense	0.2 (52 y)
				**c.985+3** **A** > **G**	Splice site	
03	1768	M	Asian Other	Homozygous c.197 T > G, p.(Met66Arg)	Missense	−0.1 (48 y)
04	18486	M	Asian Indian	Homozygous c.197 T > G, p.(Met66Arg)	Missense	0.3 (31 y)
05	19068	F	Asian Indian	Homozygous c.197 T > G, p.(Met66Arg)	Missense	0 (33 y)
06	19280	F	Asian Indian	Homozygous c.197 T > G, p.(Met66Arg)	Missense	0 (40 y)
07	19455	M	Asian Indian	Homozygous c.197 T > G, p.(Met66Arg)	Missense	0 (39 y)
08	19455	M	Asian Indian	Homozygous c.197 T > G, p.(Met66Arg)	Missense	0 (24 y)
09	19455	F	Asian Indian	Homozygous c.197 T > G, p.(Met66Arg)	Missense	0 (17 y)
10	19455	M	Asian Indian	Homozygous c.197 T > G, p.(Met66Arg)	Missense	0.5 (64 y)
11	20250	F	Asian Indian	Homozygous c.197 T > G, p.(Met66Arg)	Missense	1.4 (55 y)
12	20250	M	Asian Indian	Homozygous c.197 T > G, p.(Met66Arg)	Missense	0 (56 y)
13	20502	F	Asian Indian	Homozygous c.197 T > G, p.(Met66Arg)	Missense	0.2 (35 y)
14	5048	F	Asian Indian	Homozygous c.197 T > G, p.(Met66Arg)	Missense	0.3 (43 y)
15	5048	F	Asian Indian	Homozygous c.197 T > G, p.(Met66Arg)	Missense	0 (41 y)
16	28956	F	Unknown	Homozygous c.197 T > G, p.(Met66Arg)	Missense	PL (53 y)
17	28240	M	Unknown	Homozygous c.197 T > G, p.(Met66Arg)	Missense	0 (33 y)
18	19705	F	Unknown	**Homozygous c.279** **G** > A, p.(Trp93*)	Nonsense	0.2 (29 y)
19	18641	M	White other	c.283 G > A, p.(Gly95Arg)	Missense	0.06 (15 y)
				c.637_641del, p.(Ser213*)	Deletion	
20	26639	F	White other	**Homozygous c.414-1** **G** > **A**	Splice site	0.5 (29 y)
21	17557	F	Middle Eastern	Homozygous c.677 T > A, p.(Met226Lys)	Missense	0.3 (25 y)
22	18984	F	East Asian	Homozygous c.802-8_810delinsGC, del of exon 7	Deletion	0 (37 y)
23	19712	M	White other	Homozygous c.802-8_810delinsGC, del of exon 7	Deletion	-
24	4795	M	East Asian	c.802-8_810delinsGC, del of exon 7	Deletion	0.5 (28 y)
				c.1199 G > A, p.(Arg400His)	Missense	
25	22495	M	Unknown	c.802-8_810delinsGC, del of exon 7	Deletion	CF (48 y)
				c.992 A > C, p.(His331Pro)	Missense	
26	15601	F	White British	Homozygous c.998 C > A, p.(Thr333Lys)	Missense	0 (51 y)
27	27072	F	Unknown	Homozygous c.1168 C > T, p.(Arg390Cys)	Missense	0.6 (33 y)
28	18373	F	White other	Homozygous c.1393 A > G, p.(Arg465Gly)	Missense	0.5 (55 y)

Family identifier (GC number), gender (F: female, M: male), ethnicity, and best corrected visual acuity (BCVA, LogMAR) at first visit and corresponding age is also reported. Novel variants are reported in bold.

*CF* Counting fingers, *PL* Perception of light.

### SD-OCT quantitative measures

At least one SD-OCT exam was available and gradable in 24 patients (median age: 37 years, IQR: 32–53). Sixteen patients had 2 or more follow-up visits (median 3.5 visits, IQR: 2.8–7.3) for longitudinal analysis, resulting in median follow-up of 7.5 years (IQR: 2–11). At baseline, 2 patients had CMO (8.3%), and 2 had epiretinal membranes (8.3%).

**Fig. 1:**
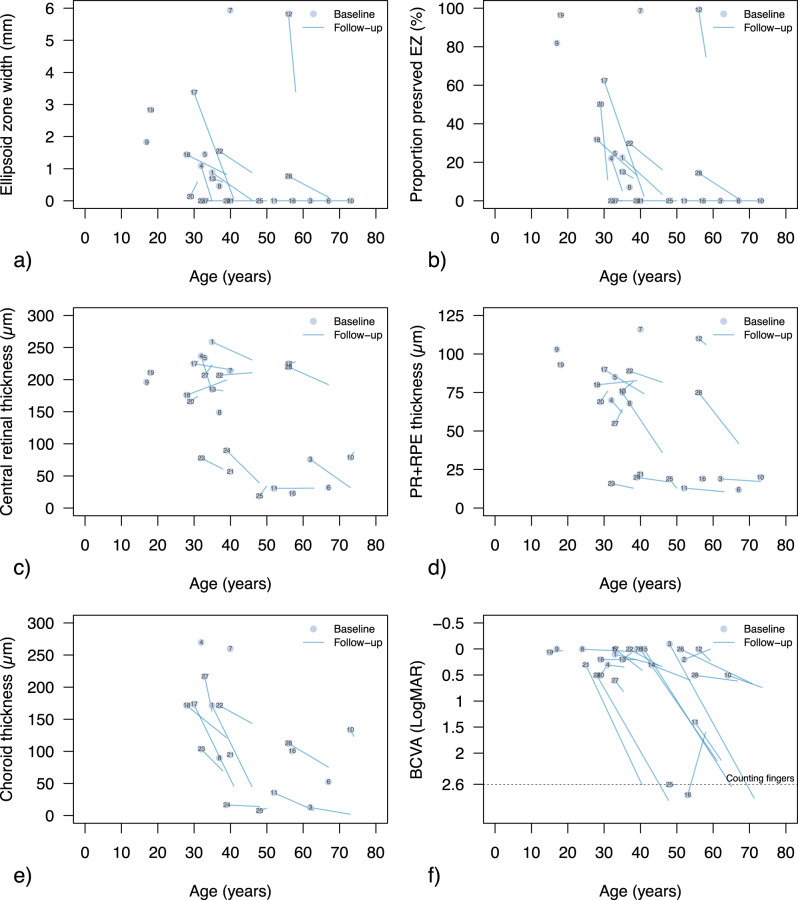
Changes in quantitative clinical measures by age. Ellipsoid zone (EZ) width and proportion-preserved are reported in (**a**, **b**), retinal thickness of the whole retina (CRT), photoreceptor and retinal pigment epithelium complex (PR + RPE) and choroid are reported in (**c**–**e**). In (**f**), data for best corrected visual acuity (BCVA) are presented. In all subplots, patients’ data at the first visit are reported as data points and the patient ID as per Table 1 is also reported. For patients with follow-up visits, the best linear fit for quantitative measures at all visits was computed and it is reported for each patient (segments). The dashed line in (**f**) reports BCVA level for counting fingers.

Ten patients (41.7%) showed severe outer retinal disruption with no measurable EZ at baseline. In patients with a measurable EZ, the median width and proportion-preserved were 1447.4 µm (IQR: 797.6–2580) and 30.8% (IQR: 22.2–77.0), respectively. At the last visit 9 (56.3%) patients showed severe disruption, with only 6 (37.5%) patients having a measurable EZ width (median 588.0 µm, IQR: 522.1–679.0), and 7 patients (43.75%) having a measurable proportion-preserved EZ (median 10.9%, IQR: 7.9–11.8). Effect of age on EZ measures is reported in Table 2 and Fig. 1. Measures at baseline exhibited substantial inter-subject variability, and there was no significant age-effect on both EZ parameters (both *p* > 0.05). In contrast, longitudinal analysis showed a significant age effect on EZ width and proportion-preserved EZ (both *p* < 0.001), with a rate of loss of −90.35 µm/year (95%CI: −134.6 to −46.09) and −3.31%/year (95%CI: −6.06 to −0.56).

**Table 2:** Age-related changes and progression rates of quantitative clinical measures in patients with Bietti Crystalline Distrophy in this cohort.

*Measure*	*Cross sectional analysis*				*Longitudinal analysis*			
	Estimate (95% CI)	*p*-value	R^2^	Adj R^2^	Estimate (95% CI)	*p*-value	Marg R^2^	Cond R^2^
EZ width (µm/year) N_Cross_: 14; N_Long_: 9	43.20 (−56.7 to 143.1)	0.36	0.068	−0.01	−90.35 (−134.6 to −46.09)	**0.0003**	0.216	0.947
Proportion-preserved EZ (%/year) N_Cross_: 14; N_Long_: 9	−0.47 (−2.36 to 1.41)	0.59	0.024	−0.06	−3.31 (−6.06 to −0.56)	**<** **0.0001**	0.234	0.995
CRT (µm/year) N_Cross_: 22; N_Long_: 15	−3.01 (−5.06 to −0.96)	**0.006**	0.319	0.285	−2.04 (−3.73 to −0.35)	**0.021**	0.089	0.992
PR + RPE (µm/year) N_Cross_: 22; N_Long_: 15	−1.35 (−2.24 to −0.45)	**0.005**	0.328	0.295	−1.15 (−1.73 to −0.58)	**0.0002**	0.218	0.923
Choroid (µm/year) N_Cross_: 18; N_Long_: 13	−2.94 (−5.65 to −0.23)	**0.036**	0.248	0.201	−5.40 (−8.15 to −2.65)	**<** **0.0001**	0.427	0.992
BCVA (LogMAR/year) N_Cross_: 25; N_Long_: 23	0.007(−0.004 to 0.018)	0.19	0.074	0.034	0.016(0.006 to 0.027)	**0.0019**	0.252	0.790

Statistically significant age-related changes (*p* < 0.05) are reported in bold.

*EZ* Ellipsoid zone, *CRT* Central retinal thickness, *PR* *+* *RPE* Photoreceptor and retinal pigment epithelium complex, *BCVA* Best corrected visual acuity. N_Cross_ and N_Long_: number of patients with usable data for cross-sectional and for longitudinal analysis, respectively. *Adj R*^*2*^ Adjusted R^2^, *Marg R*^*2*^ Marginal R^2^, *Cond R*^*2*^ Conditional R^2^.

Median CRT at baseline was 180.5 µm (IQR: 77.5–215.5), and this remained similar at the last visit (median 183.5 µm, IQR: 56–213.3). PR + RPE thickness dropped from a median 69.5 µm (IQR: 19.8–86) at baseline, to 41.5 µm (IQR: 16.8–76.8) at last visit. Age-related changes for CRT and PR + RPE were assessed after removing data from patients with CMO, and both cross-sectional and longitudinal analysis showed a small but significant age effect (all *p* < 0.05, Table 2). Longitudinal analysis showed a thinning rate of −2.04 µm/year (95%CI: −3.73 to −0.35) and −1.15 µm/year (95%CI: −1.73 to −0.58) for CRT and PR + RPE, respectively.

Choroidal thickness was measurable at baseline in 18 patients (75%). These patients were older compared to those with unidentifiable choroid-scleral junction (median age: 39.5 years, IQR: 33.5–55 vs 31 years, IQR: 20.8–34.5, *p* = 0.05) and with more severe proportion-preserves EZ loss (16.0%, IQR: 0–22.4% vs 61%, IQR: 31.0–92.7%, *p* = 0.006). At baseline, patients had median choroidal thickness of 108.5 µm (IQR: 62.3–172). This dropped to 70 µm (IQR: 12–127, *n* = 13) at last visit, after a median 9 years (IQR: 3–11). There was significant choroid thinning with age, with large rate of loss (−5.40 µm/year, 95%CI: −8.15 to −2.65, *p* < 0.0001, Fig. 1, Table 2). Enhanced-depth-imaging (EDI) scans were available in 3 patients and were not considered for analysis.

### Ocular imaging

Sixteen patients had available pseudo-colour fundus photos (57.1%, median age 43 years, IQR: 36.5–56; Supplementary Table 1), and majority of patients presented the more advanced BCD stage 3 (*n* = 12, 75%). Median BCVA was 0.25 LogMAR (IQR 0.125–0.375), 0.3 LogMAR (IQR 0.15–0.45), and 0.75 LogMAR (IQR 0.2–2.63) at BCD stages 1 to 3. Similarly, proportion-preserved EZ measures showed increasing disruption with increasing BCD severity (median 66.0%, IQR: 58.1–73.9; 49.6%, IQR: 24.8–74.4; and 1.9%, IQR: 0–11.4 at stage 1 to 3). Nonetheless, differences by severity were not statistically significant (both *p* > 0.05).

**Fig. 2:**
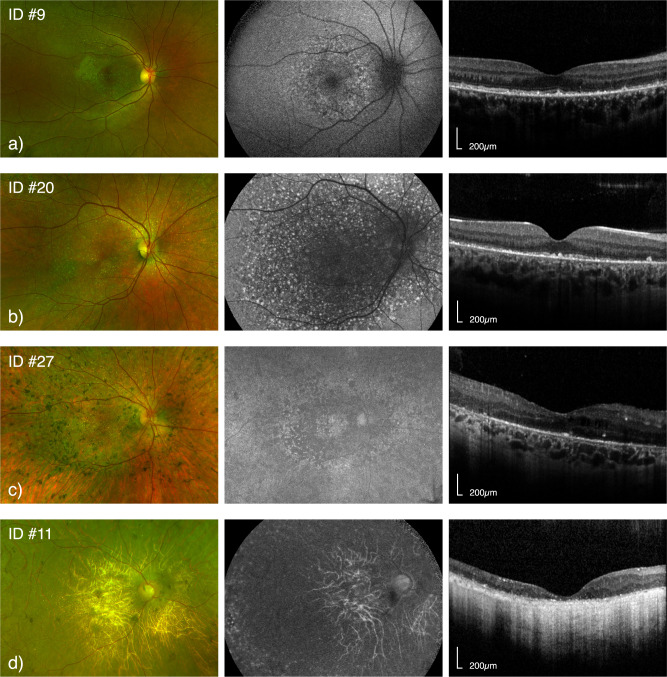
Pseudo-colour fundus photo, fundus autofluorescence (FAF) and foveal OCT scans for 4 patients with Bietti Crystalline Dystrophy. From (**a**–**e**) patients with increasing age and severity are reported. In (**a**): 17 year old patient with BCVA of 0.0 LogMAR at the same age. In (**b**) a 29 year old patient with BCVA of 0.5 LogMAR at the time if imaging. In (**c**) a 31 year old patient with BCVA of 0.6 LogMAR at age 33. Lastly, (**d**) shows a 56 years old patient with BCVA of hand movements. For each patient, FAF and OCT foveal scan were extracted at the same time point of the fundus photo.

FAF was obtained and gradable in in 23 patients (82%; Supplementary Table 1). All patients showed FAF abnormalities at baseline, and we found significantly poorer BCVA at increasing FAF severity (*p* = 0.012), with median BCVA dropping from 0.3 LogMAR (IQR: 0–0.55) in patients with posterior pole changes to 1.4 LogMAR (IQR: 0.8–2.6) in patients with severe FAF loss. Consistently with OCT findings, patients with severe FAF loss had complete EZ atrophy, whereas patients with FAF changes within and beyond posterior pole had median proportion-preserved EZ of 50.2% (IQR: 11.0–90.2) and 14.4% (IQR: 1.8–23.6), respectively (*p* = 0.025). Figure 2 shows examples of multimodal imaging in 4 patients.

### BCVA

BCVA was available in 27 patients (96%, median age 37 years, IQR: 30–49.5), and 26 patients had 2 or more visits with median follow-up of 7.7 years (IQR: 3.4–14.5). Median BCVA at baseline was 0.2 LogMAR (IQR: 0–0.5), dropping to 0.5 LogMAR (IQR: 0.2–2.6) at last visit. As reported in Fig. 1, BCVA showed large inter-subject variability and we did not find an age-effect while considering baseline visits only (*p* = 0.19, Table 2). Conversely, there were significant age-related BCVA changes in longitudinal analysis (*p* = 0.0019), despite at a slow rate (0.016 LogMAR/year, 95%CI: −0.006 to 0.027).

### Additional analyses

**Fig. 3:**
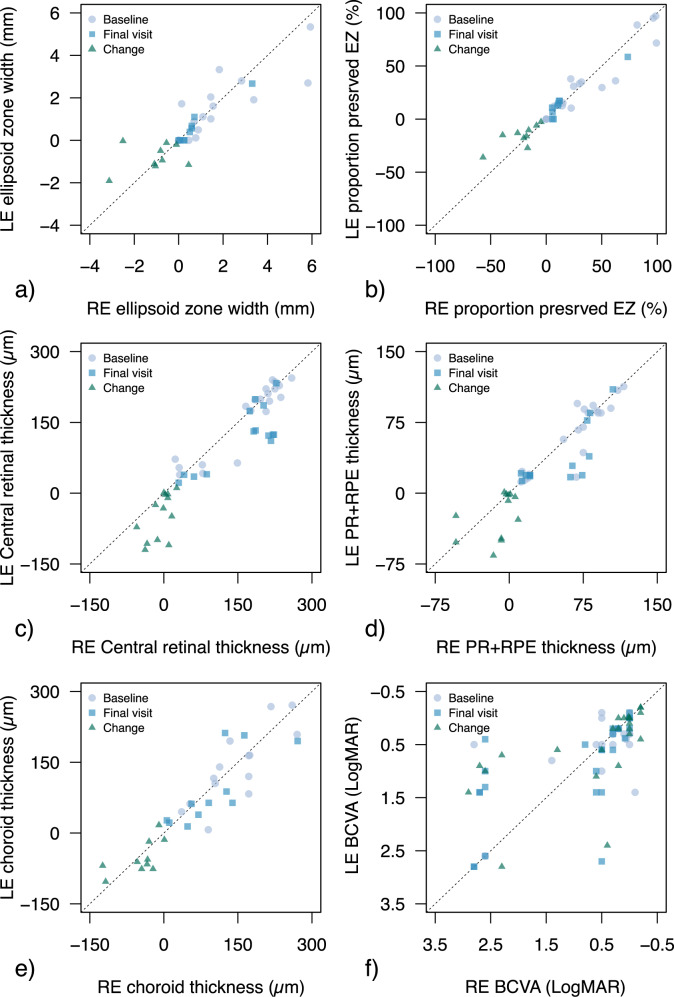
Inter-ocular relationship between quantitative clinical measures of right (RE) and left eyes (LE) at baseline, last visit and between changes from baseline. Ellipsoid zone (EZ) width and proportion-preserved are reported in (**a**, **b**), retinal thickness of the whole retina (CRT), photoreceptor and retinal pigment epithelium complex (PR + RPE) and choroid are reported in (**c**–**e**). In (**f**) we reported data for best corrected visual acuity (BCVA). A 1:1 line is also reported.

Inter-ocular relationship analysis is reported in Fig. 3 and Supplementary Tables 2. EZ measures showed strong inter-ocular correlation at baseline and last visit (*ρ*: 0.92–0.96 and 0.87–0.94, respectively). Compared to EZ width, changes from baseline of proportion-preserved EZ were also strongly related (*ρ*: 0.22 vs 0.70). All retinal thickness parameters had strong inter-ocular relationships at baseline (*ρ*: 0.84–0.88). Choroidal thickness had slightly stronger correlation at last visit compared to CRT and PR + RPE (*ρ*: 0.83 vs 0.56–0.72), whereas all thickness measures had slightly poorer correlation for changes from baseline (*ρ*: 0.50–0.66). BCVA showed moderate inter-ocular correlation at baseline, whereas it was stronger for last visit and changes from baseline ((*ρ*: 0.42 vs 0.86–0.78).

Relationship between different OCT measures at baseline and BCVA was significant for all parameters (all *p* < 0.003, Supplementary Fig. 3). OCT measures had significant correlations between themselves (*ρ*: 0.69-0.96). EZ width and proportion-preserved were strongly related (*ρ*: 0.96, *p* < 0.0001). Among retinal thickness measures, PR + RPE showed the strongest relationship with EZ parameters (*ρ*: 0.93–0.95). Relationships between BCVA and all OCT measures were also significant (*ρ* range: 0.72–0.93), with EZ parameters and PR + RPE thickness showing the strongest correlation (*ρ*: 0.88–0.93).

Phenotypic variability was seen in patients with the same variant suggesting a lack of genotype-phenotype correlation. We compared patients with the c.197 T > G vs c.802-8_810delinsGC vs any other variant, and although those with the c.802-8_810delinsGC appeared to develop a more severe phenotype, differences were not statistically significant (Supplementary Fig. 4).

There were 6 patients who developed an outcome of sight impairment (BCVA ≥ 1.0 LogMAR) over follow-up for survival analysis, with median survival time of 68.5 years (Supplementary Fig. 5).

## Discussion

We reported the longitudinal retinal structure-function changes in a large patient cohort with BCD aiding prognostic indication and future clinical trial design. Despite an understanding of the disease stages of BCD [8, 9, 13–15], little is known about age-related changes as most studies are qualitative or cross-sectional [2]. Patients in this cohort presented with initial symptoms between the age of 25 and 40 years, followed by a slow progressive degeneration, consistent with previous studies [8, 11, 12]. By age 60, most showed severe chorioretinal atrophy, and median survival time for sight impairment was 68.5 years. We found small but significant age-related changes across all clinical measures, although there was high inter-subject variability at similar ages, evidenced by poor or no age-related relationships when considering baseline visits only. In contrast, the longitudinal analysis accounted for variability at baseline and found a significant age-effect for all parameters studied.

Only a few studies provide quantitative measures of structural-functional parameters in BCD. BCVA is most commonly used as functional measurement in previous literature, and consistently with our study, this was shown to have large inter-subject variability [9, 14, 15, 21, 22]. Available studies and our results suggest the absence of a clear trend for poorer VA with increasing age when considering cross-sectional data [9, 14, 15, 21, 22]. We found an estimated BCVA loss of +0.016 LogMAR per year (95%CI: 0.006-0.027). Another longitudinal study by Murakami et al. assessed 55 East Asian patients with BCD and found a VA loss of +0.089 LogMAR/year [21]. Most of their patients had well-preserved baseline BCVA (mean: 0.29 LogMAR) at relatively advanced age (mean: 55.2 years) [21]. Their more rapid rate of BCVA loss could be explained by the baseline vision and advanced age of Murakami’s cohort.

Structural measures of the EZ and choroid showed the fastest progression rates and could be preferable for monitoring BCD. Although there are no reports for EZ parameters in BCD, previous studies assessed choroidal thickness in EDI OCT scans and found large differences compared to healthy controls with increasing BCD severity [9, 36]. While acknowledging the limitations of choroidal visualisation in non-EDI imaging, our findings are consistent with those studies [9, 36]. Although our choroidal thickness appeared slightly lower compared to previously reported, differences could be attributed to different imaging modalities, with standard scans being progressively less accurate with a thicker choroid [37]. Also, patients whose choroid-scleral junction could not be identified in our setting had more preserved outer retina, suggesting an underestimation of average choroidal thickness in our sample. Overall, results suggest that the choroid may be a valuable structure for monitoring BCD, and emphasizes the importance of imaging methods able to enhance choroidal visualisation.

### Genetic findings

There were 15 *CYP4V2* variants in our cohort, with the most frequent variants being c.197 T > G and c.802-8_810delinsGC. Consistently with the majority of published BCD studies [8–11, 14, 21, 38], we found substantial intra and inter-familial phenotypic variability (Supplementary Fig. 4), suggesting that epigenetic or environmental factors may influence lipid metabolism and disease expression. Notably, some studies proposed the c.802-8_810delinsGC variant to result in more severe phenotype [8, 10, 22], however, not achieving statistical significance. Similarly, our structural and functional parameters appeared more affected with the c.802-8_810delinsGC variant, without reaching significance. In their large study, Murakami et al. [21] found no difference in BCVA deterioration between patients with c.802-8_810delinsGC (*n* = 33) and other variants (*n* = 22). Additional studies may be required to fully understand if any genotype-phenotype correlations exist, ideally including larger samples with heterogeneous representation of mutations and ethnicities, and considering suitable structural parameters.

### Consideration for future clinical trials

Novel BCD treatments are currently explored pre-clinically [1–3], and our findings can help with clinical trial study design. The ideal intervention window appears difficult to be solely defined by age, due to the large inter-individual variability. We found a large lag between symptom onset (age 25–40) and the age when most patients showed severe atrophy (age 60–65). It seems plausible to administer treatment after symptom onset, but the intervention window should perhaps be guided by clinical findings, such as level of preserved EZ and choroidal parameters.

In considering potential outcome metrics, BCVA was a poor parameter for BCD monitoring due to its slow age-related loss. Also, as suggested by interocular correlation and structure-function analyses, BCVA may not capture disease stage accurately as severe disease may coexist with relatively well-preserved BCVA if structural remnants are present at the fovea.

Choroidal thickness and proportion-preserved EZ were better outcome metrics in our cohort, with faster progression rates compared to other measures (2.3–3.3%/year vs 0.6–1.5%/year; rates normalised for their dynamic ranges). We extracted two measures of EZ; width and proportion-preserved. In BCD, EZ width may overestimate disease severity if focal defects are present near the fovea with preserved EZ on either side. Indeed, relatively young individuals with early BCD could present with reduced EZ width due to such central focal defects (Fig. 2a, b). To account for these localised changes, we measured proportion-preserved EZ which provided a more representative parameter of disease status in some participants, by capturing preserved EZ across the whole foveal B-scan (e.g., patient ID #9, #19 and #20, Fig. 1a, b). These differences suggest that future BCD metrics should capture focal changes, but also be robust to the heterogeneous retinal modifications. This appears in agreement with findings by Liu et al. [15] which showed stronger inter-ocular relationships for mean retinal thickness across volumetric scans compared to foveal thickness (r: 0.73–0.76 vs 0.49).

A key BCD feature is retinal crystals. Available studies report crystal formation as an early sign of disease, and their disappearance with disease progression [8, 15]. Accordingly, crystals may be absent in a healthy retina as well as in a completely atrophic retina, and this attribute may represent a serious drawback for their consideration as an outcome metric.

The main limitation of this study was the retrospective nature, resulting in variable types and numbers of examinations/retinal imaging between patients, with different time scales. Investigations which would be relevant for monitoring BCD patients, such as perimetry and electrophysiology, were not available for the majority of patients for follow-up analyses. OCT measures were taken over the foveal B-scan, however, the volumetric scan provides more information than a single scan approach, which may fail to capture non-uniformities in the pattern of retinal changes [39]. Changes in FAF were only assessed qualitatively as all patients had non-standardised FAF imaging as part of their routine clinical care (e.g. differing FAF field of view and some scans were affected by poor centration). This limited our capability of conducting a quantitative analysis, hence future studies in BCD should aim to quantitatively assess FAF changes over time. Lastly, quantitative imaging measures were extracted by one single senior grader at a single time point. Although these measures are established for inherited retinal disease, with accepted inter- and intra-observer reliability [24, 29, 40], automated extraction may improve the data collected.

Overall, this study provides a quantitative analysis of BCD natural history. Affected individuals report their first symptoms between age 25 and 40 years, with slow progression to advanced chorioretinal atrophy and severe vision loss by age 60. BCD patients showed phenotypic variability with no clear genotype-phenotype correlation. BCVA may be preserved until late in the disease course, and may not be representative of disease progression. OCT parameters capturing global changes of EZ and choroid may be more suitable outcome measures for future clinical trials; they should be further examined in larger prospective patient studies.

## Summary

### What was known before

- Bietti Crystalline Dystrophy (BCD) is a progressive inherited retinal disorder resulting in dysfunctional lipid metabolism and atrophy of retinal pigment epithelium. BCD presents between age 25 and 40 with reduced vision and night blindness, and slowly progresses to an advanced chorioretinal atrophy and vision loss by the 5th or 6th decade of life.

### What this study adds

- We have characterised the natural history of patients with BCD to identify potential outcome metrics for future clinical trials. Visual acuity may be preserved until late, and seemed poorly representative of disease progression. OCT parameters capturing changes of the ellipsoid zone and the choroid may afford more suitable trial outcome measures.

### Supplementary information

Supplementary Material

Supplementary File 1

## Data Availability

The raw and summarised data presented in this study are reported in the main tables/figures and in the supplementary materials. Full datasets and customised algorithms for image analysis are available on request from the corresponding author.
