# Supplementary material for: Longitudinal structure-function analysis of molecularly-confirmed CYP4V2 Bietti Crystalline Dystrophy
Source: Eye (Lond). 2023 Oct 28;38(5):853–62. doi: 10.1038/s41433-023-02791-7 (PMC10966004; doi:10.1038/s41433-023-02791-7)
Supplement: Supplementary file 1 — Supplementary Material [file 41433_2023_2791_MOESM1_ESM.pdf]

Supplementary Material

Longitudinal structure-function analysis of molecularly-confirmed CYP4V2 Bietti Crystalline Dystrophy.

Supplementary Tables

Supplementary Table 1. Clinical characteristics and demographics of patients with Bietti Crystalline Dystrophy in this study. Continuous variables are reported as median and interquartile range (IQR); categorical variables as count (n) and frequency (%).

	n or median	% or IQR
**Gender** (female / male)	16 / 12	57.1% / 42.9%
**Ethnicity**		
Asian Indian, n (%)	12	42.9%
East Asian, n (%)	2	7.1%
Asian Other, n (%)	1	3.6%
Middle Eastern, n (%)	1	3.6%
White (any), n (%)	7	25.0%
Unknown, n (%)	5	17.9%
**Age at first symptoms** (n=18) Median (IQR), years	29	25-38
**Presenting symptoms** (n=25)		
Night Blindness, n (%)	14	56.0%
Peripheral Vision Loss, n (%)	7	28.0%
Central Vision Loss, n (%)	5	20.0%
Metamorphopsia, n (%)	3	12.0%
Photophobia, n (%)	3	12.0%
Photo-attraction, n(%)	2	8.0%
Asymptomatic, n (%)	4	16.0%
**Age at first visit** (n=27) Median (IQR), years	37	30-49.5
**Age at last visit** (n=26) Median (IQR), years	46.8	38.9-60.6
**BCVA baseline, RE** (n=27) Median (IQR), LogMAR	0.2	0-0.5
**BCVA last visit, RE** (n=26) Median (IQR), LogMAR	0.5	0.2-2.6
**Fundus photo baseline (Yuzawa et al stages), RE** (n=16)		
Stage 1 (RPE atrophy and crystalline deposits in the macular area), n (%)	2	12.5%
Stage 2 (RPE atrophy beyond macula. Choroid atrophy at macula. Less crystals in areas with advanced atrophy), n (%)	2	12.5%
Stage 3 (Advanced RPE and choroid atrophy beyond macula) n (%)	12	75%
**FAF baseline, RE** (n=23)		
Normal FAF, n (%)	0	0%
FAF changes limited to posterior pole, n (%)	7	30.4 %
FAF changes beyond posterior pole, n(%)	11	47.8%
Severe-complete FAF loss, n(%)	5	21.7%
**OCT metrics baseline, RE** (n=24)		
Qualitative		
Intact EZ & RPE, n (%)	0	0%
Focal EZ loss at the macula, n (%)	6	25%
Focal EZ & RPE residual at the macula, n (%)	8	33.3%
Severe disruption, n (%)	10	41.7%
Quantitative at baseline		
EZ width (n=14, with preserved EZ), µm	1447.4	797.6-2580
Proportion-preserved EZ (n=14 with preserved EZ), %	30.8	22.2-77.0
CRT (n=24), µm	180.5	77.5-215.5
PR+RPE thickness (n=24), µm	69.5	19.8-86
Choroid thickness (n=18), µm	108.5	62.25-172
**Corneal examination,** (n=27)		
Crystals, n (%)	9	33.3%
Clear, n (%)	2	7.4%
Info not available, n (%)	16	59.3%

BCVA: best corrected visual acuity; RE: right eye; FAF: fundus autofluorescence; EZ: ellipsoid zone; CRT: central retinal thickness; PR+RPE: photoreceptor and retinal pigment epithelium complex.

Supplementary Table 2. Inter-ocular correlation (Spearman's rho, and 95% confidence interval) for quantitative clinical measures considered.
EZ = Ellipsoid zone; CRT = Central retinal thickness; PR+RPE = Photoreceptor and retinal pigment epithelium complex; BCVA = Best corrected visual acuity.

Clinical Measures	Baseline	Last visit	Change from baseline
EZ width	0.92 (0.80 to 0.97)<br>p<0.0001	0.96 (0.81 to 0.99)<br>p<0.0001	0.22 (-0.52 to 0.77)<br>p=0.58
Proportion-preserved EZ	0.94 (0.86 to 0.98)<br>p<0.0001	0.87 (0.47 to 0.97)<br>p=0.003	0.70 (0.07 to 0.93)<br>p=0.04
CRT	0.88 (0.71 to 0.95)<br>p<0.0001	0.56 (0.05 to 0.84)<br>p=0.04	0.50 (-0.08 to 0.82)<br>p=0.09
PR+RPE	0.84 (0.64 to 0.94)<br>p<0.0001	0.72 (0.28 to 0.91)<br>p=0.006	0.52 (-0.08 to 0.84)<br>p=0.08
Choroid	0.85 (0.59 to 0.95)<br>p=0.0001	0.83 (0.47 to 0.96)<br>p=0.001	0.66 (0.06 to 0.91)<br>p=0.04
BCVA	0.42 (0.05 to 0.69)<br>p=0.029	0.86 (0.70 to 0.93)<br>p<0.0001	0.78 (0.60 to 0.90)<br>p<0.0001

Supplementary Figures

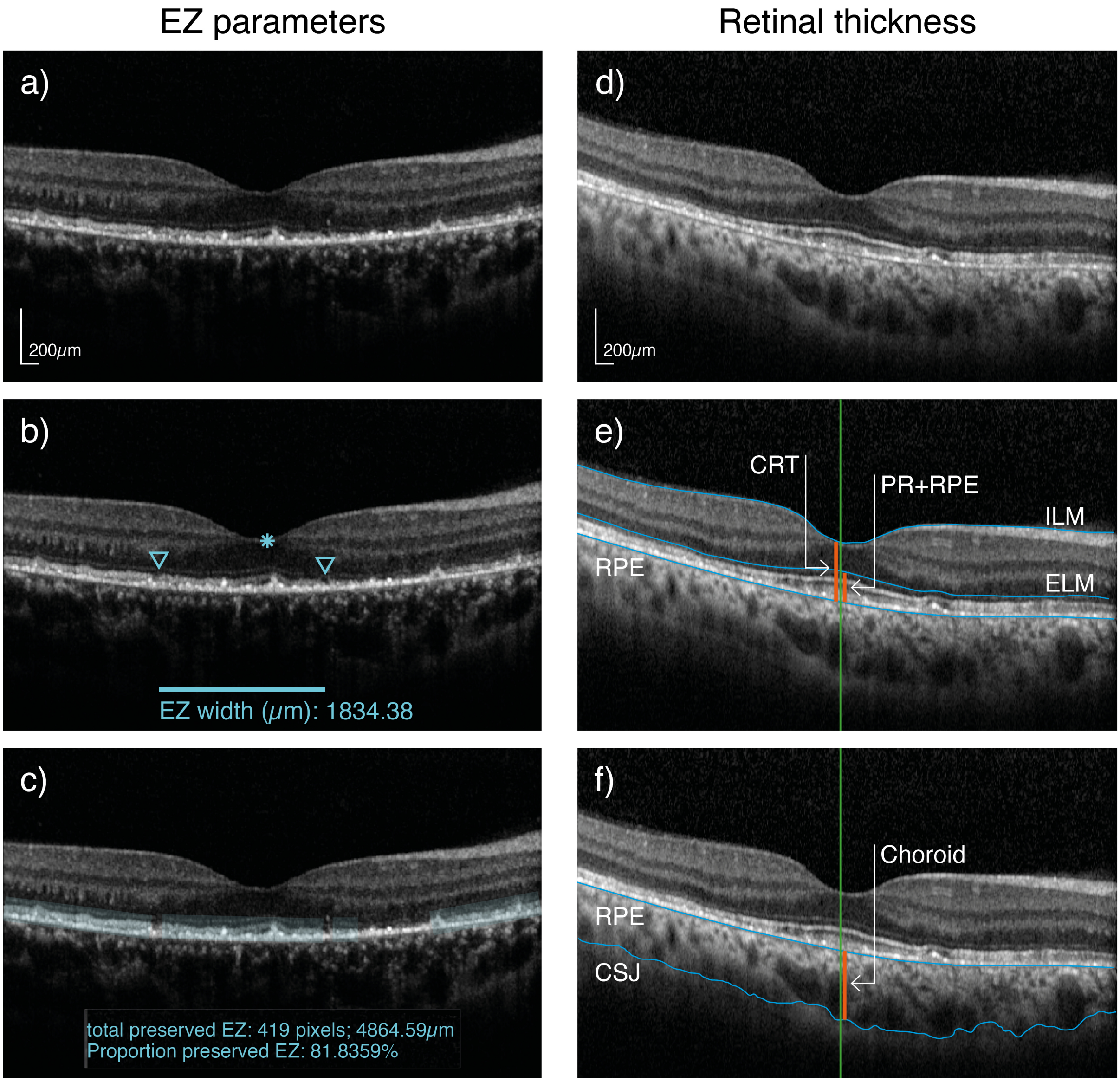

Supplementary Figure 1: Details of quantitative OCT measures considered in this study, for ellipsoid zone metrics (a, b, c) and retinal thickness (d, e, f), in two included participants. Quantitative OCT metrics were extracted along the horizontal foveal B-scan, identified as the B-scan having the least residual inner retinal tissue and thickest outer nuclear layer. Foveal B-scan were extracted from Spectralis (a) and read in MATLAB alongside the corresponding lateral resolution (μm/pixel). Firstly, the fovea was identified, and then EZ width was measured as the lateral distance between the first EZ interruption nasally and temporally from the fovea (b). Proportion-preserved was measured by identifying all preserved EZ segments in the foveal B-scan. Then the cumulative sum of preserved EZ was divided by B-scan width to obtain % measure (c). All patients but one were imaged with 20deg-wide scan protocols. In the remaining patient a 30deg-wide cube was used, and for consistency between EZ measures, foveal B-scans from this patient were cropped to 20deg. Retinal thickness was measured at the fovea along the green line in (e, f) as vertical distance between: inner limiting membrane (ILM) and retinal pigment epithelium (RPE) for central retinal thickness (CRT, e); between external limiting membrane (ELM) and RPE for photoreceptor and RPE complex (PR+RPE, e), and between RPE and choroidal-scleral junction (CSJ) for choroid (f). All grading tasks were conducted according to written protocol, on a 21.5" LED monitor (Dell Technologies, Round Rock, Texas, US) in standardised settings.

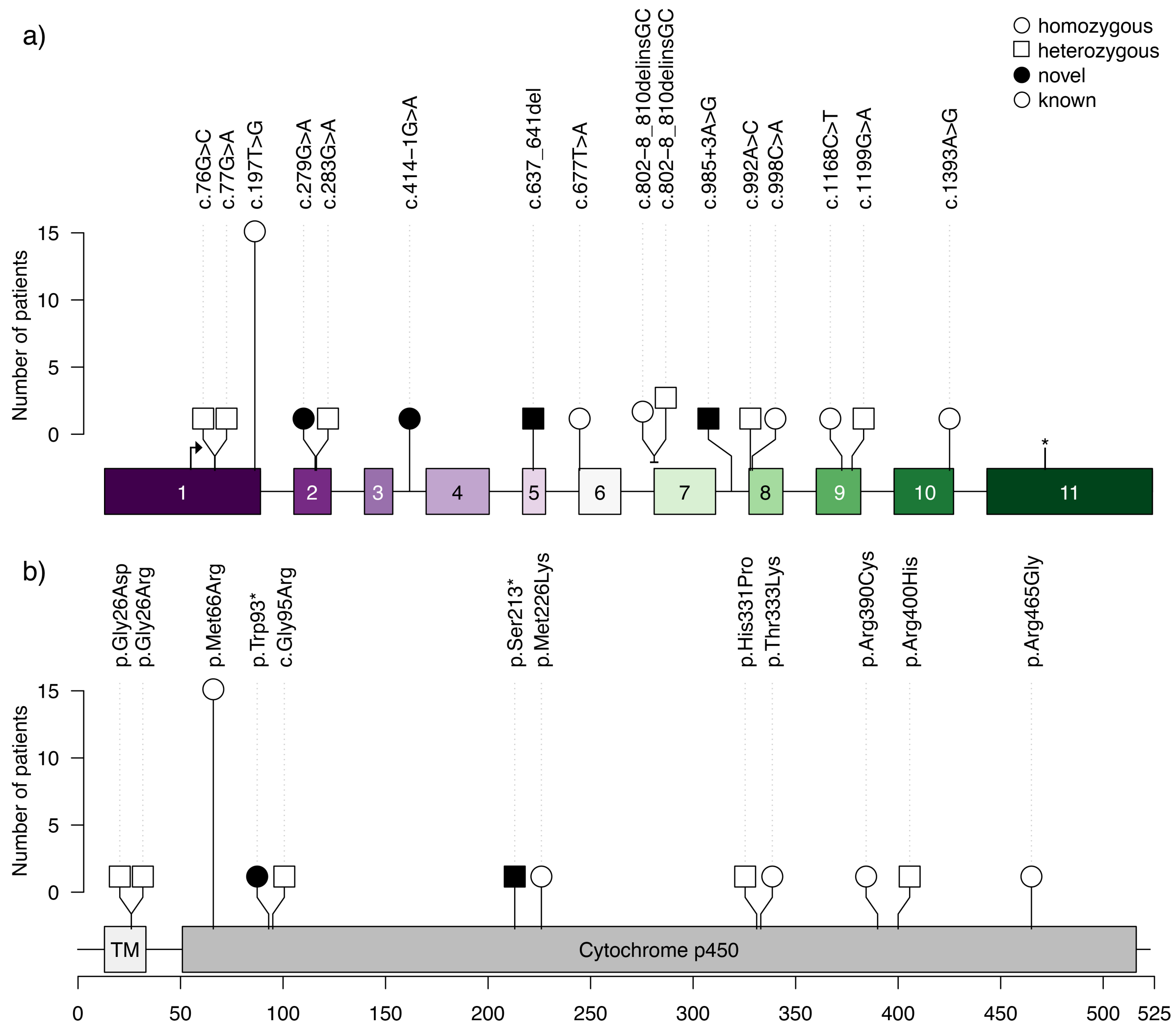

Supplementary Figure 2: Details and positions of *CYP4V2* variants in this cohort and corresponding amino-acid changes. A schematic diagram of *CYP4V2* gene is reported in a (exon 11 is not reported in scale for representation purpose). Genetic variants are reported as lollipops at the corresponding position, and colour and shape-coded according to novelty and status. In b, a schematic diagram of *CYP4V2* protein structure is reported, with its 2 domains (TM = transmembrane).

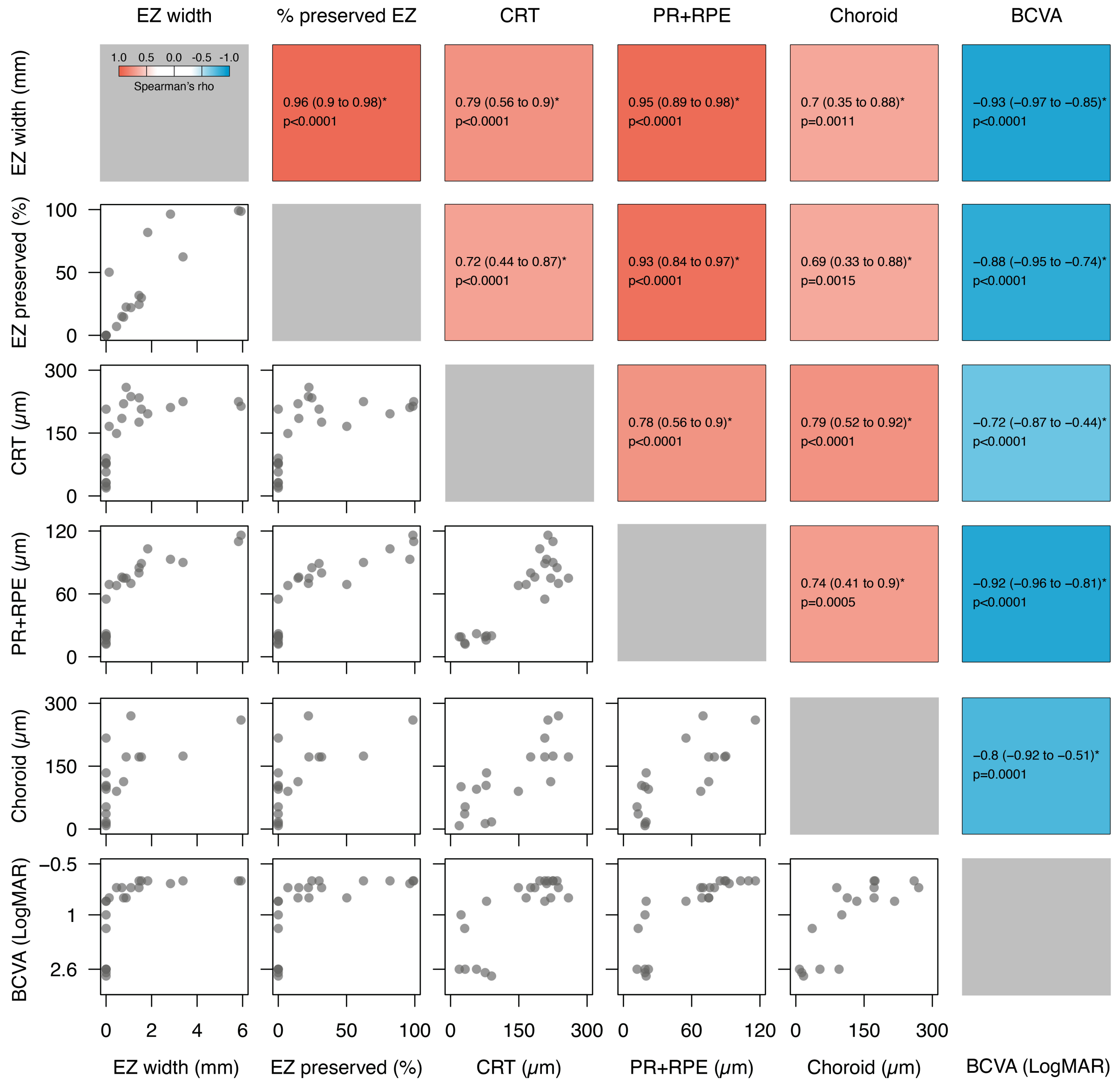

Supplementary Figure 3: Scatterplots showing the relationship between different OCT measures and best corrected visual acuity (BCVA). In the right hand-side of the plot, Spearman's correlation coefficients and their 95% confidence intervals are also reported and colour-coded according to strength of correlation. Data for baseline OCT examination were considered, and VA values were reported at the closest age. After Bonferroni correction (15 comparisons), correlation was considered to be significant if p<0.003 and flagged with *.

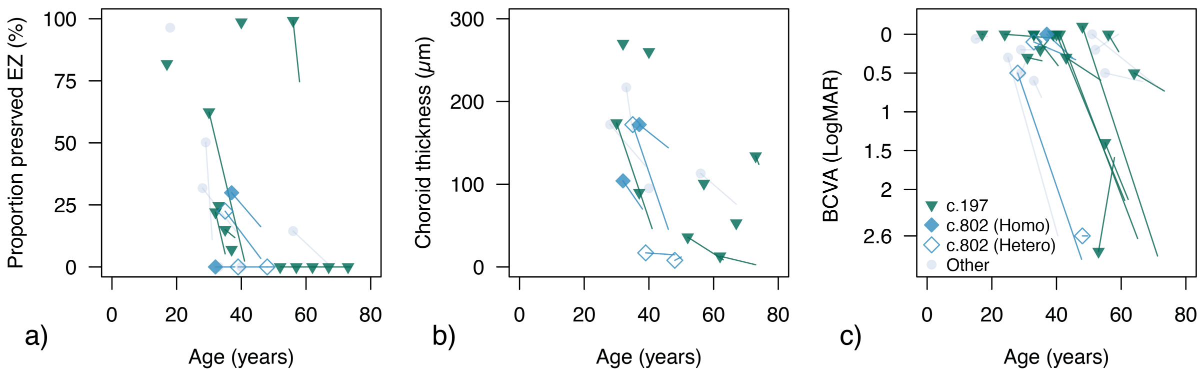

Supplementary Figure 4: Differences in proportion-preserved ellipsoid zone (a), choroid thickness (b) and best corrected visual acuity (BCVA, c) by genetic variants. Genotype was classified according to most frequent variants in our sample, in patients with c.197T>G (p.Met66Arg) mutations (c.197), c.802-8_810delinsGC (c.802) mutations and any other, and data points are coded accordingly. Consistently with Figure 1, both baseline visit (single data point) and progression rate (segments) are reported for each individual patient. Only proportion-preserved EZ and choroid thickness were reported as OCT metrics as the one showing more substantial age-related changes in previous analysis (see results). Data seemed to suggest patients with c.802-8_810delinsGC variants to have more severe phenotype compared to c.197T>G variant (proportion-preserved EZ: -28.5%, 95%CI: -57.8 to 0.8; Choroid thickness: -81.0μm, 95%C: -151.9 to -10.1; and VA +0.90 LogMAR, 95%CI: -0.24 to 2.04). However, none of the differences exceeded statistical significance (proportion-preserved EZ, p=0.13; Choroid thickness, p=0.07; BCVA, p=0.29).

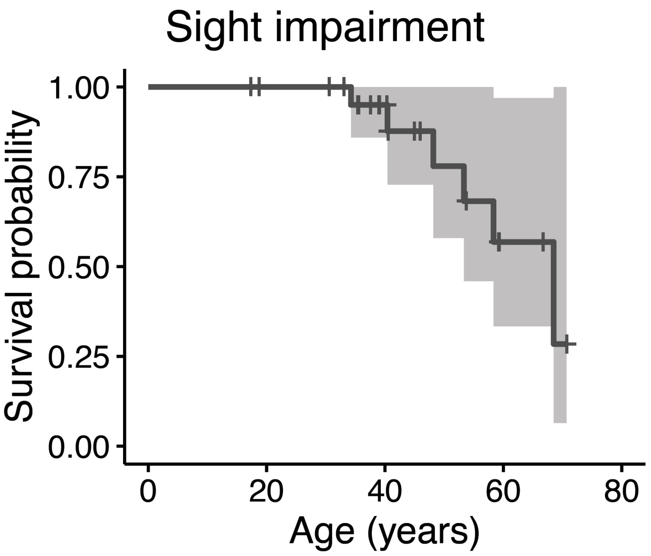

Supplementary Figure 5: Survival analysis for best corrected visual acuity (BCVA). An outcome of BCVA ≥ 1.00 LogMAR (sight impairment) was considered. 95% confidence intervals are also reported underneath survival curve.
