# Supplementary material for: Longitudinal structure-function analysis of molecularly-confirmed CYP4V2 Bietti Crystalline Dystrophy
Source: Eye (Lond). 2023 Oct 28;38(5):853–62. doi: 10.1038/s41433-023-02791-7 (PMC10966004; doi:10.1038/s41433-023-02791-7)
Supplement: Supplementary file 2 — Supplementary File 1 [file 41433_2023_2791_MOESM2_ESM.pdf]

SUPPLEMETARY FILE 1

Automated crystal count

Retinal crystals are a key feature of BCD and we explored objective and automated quantification of crystals to test suitability as outcome measure in clinical trials.

As suggested before,[1-3] our image analysis focussed on green channel for maximum contrast, then we used a smoothing filter to reduce noise and extracted crystal metrics. All image processing was performed in MATLAB (Version 9.6.0, The MathWorks Inc., Natick, MA).

Firstly, we read OPTOS (California) dicom image (*.dcm)* as extracted from software into MATLAB. Images were then converted in grey scale by considering only the green channel, which provides the best visibility of white alterations on the fundus. Left eyes were flipped to right eyes format for consistency. By using the OCT scan as guidance, we subjectively selected the fovea on the fundus photo, and also the area corresponding to a 20-by-20 degrees square. This enabled us to identify a conversion from OPTOS pixel to degrees. Images from each patient were then cropped to include a 30-by-30 degrees square centred onto the fovea, enabling full inclusion of the ETDRS circles. This was accomplished by selecting corresponding points in OPTOS and OCT SLO image at the border of the scan, enabling to extract corresponding distance of scan width in the OPTOS fundus photo.

Image processing was then applied to optimise crystal count:

- Smoothing filter to reduce noise (3x3 Wiener Filter);
- Determine the background of the image (eye and visit specific), by using *imopen* and *strel* functions in MATLAB and considering disk-shaped structuring element with a radius of 3 pixels. This process aims to remove elements in the image with dimensions within the chosen radius value, and replacing them with an estimate of background intensity. As reported in Figure 1, a value of 3 pixels was consistent with crystal size and provided best result without emphasising larger retinal details (i.e., blood vessels).

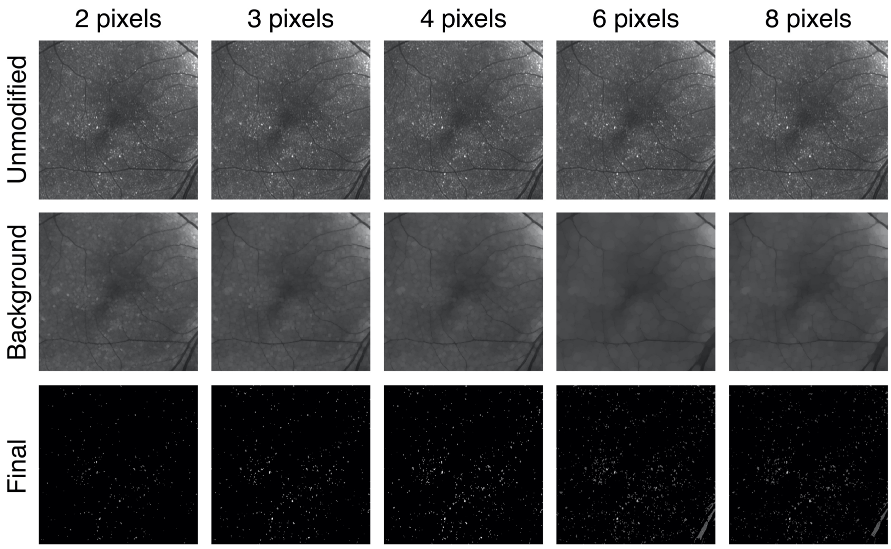

Figure 1

Differences in background extraction with disk-shaped structuring elements of increasing radius. Top panel shows the unmodified grey-scale image. The background extracted after disc-shaped structure removed is reported in the middle panel. The bottom panel reports the result of image division (unmodified image divided by background), emphasising disk-shaped elements in the original image.

- Grey scale images were then divided by the background image and a threshold transformation was applied to dichotomise images (intensity >1 was considered as threshold).
- Consideration of the area to assess required conversion from pixel to µm or degrees. Providing a precise value in degrees or µm has limitation within OPTOS images, due to varying aspect ratios moving from the centre to the periphery. Accordingly, images may not be symmetric but rather distorted.[4,5] However, measures taken at the posterior pole should be less affected by distortions, with studies suggesting that aspect ratio could be close to 1 within the macula.[4] We used Spectralis resolution to guide conversion from OPTOS pixel to µm/mm.

In patients presenting multiple examinations, follow-up images underwent registration to baseline before any additional analysis. Image registration to the baseline image (first visit for the specific patient) was performed to ensure consistent examinations with follow-up and crystal count. This was achieved via the MATLAB's built-in control points selection tool (cpSelect function). Coordinates of corresponding points were extracted from the baseline fundus photo and the follow-up image. We manually selected a minimum of 9 points representing the same retinal detail (e.g. a blood-vessels intersection). The identified points were then used to compute a geometric projective transformation that align image at follow-up with that at baseline. After registration, follow-up images underwent the same processing described above.

The quantitative image metrics considered were the number of crystals within 3 ETDRS circles (1, 3, and 6mm diameter), centred on the fovea. Focus on these three concentric circles, named foveal centre, inner macular ring and outer macular ring, enabled us to reduce impact of blood vessels, and also the optic disc. The distance from fovea and the size of each crystal were also extracted.

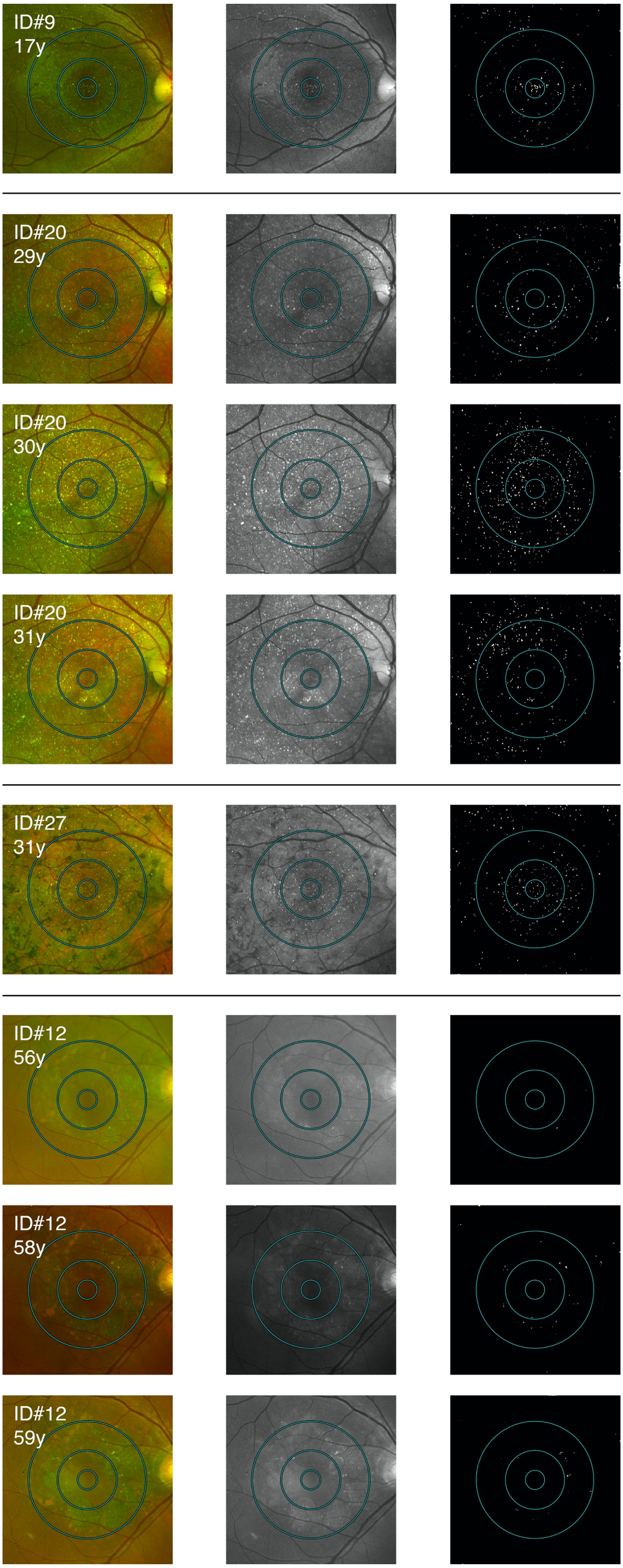

Figure 2

Example of final images in patients with BCD. The left panel reports 30-by30 degrees crops in original pseudo-colours. The green channel only is reported in the middle panel, and the final transformation for crystal count with ETDRS circles overlapped is reported in the right panel.

Only 4 eyes presented with Yuzawa's stage 1 or 2 at baseline, with majority of patients showing severe chorio-retinal atrophy and no crystal measures to extract (n=12). Also, atrophic changes resulted in artefacts in automated crystal detection, as small atrophic ramifications had similar pixel dimension with crystals (see Figure 2d from manuscript).

The summary of results of crystal count are reported in Table 1 below. Median (range) ETDRS crystal count across all eyes at their first visit were:

- 8.5 (0-17) for ETDRS 1mm,
- 51 (1-106) for ETDRS 3mm, and
- 142.5 (3-221) for ETDRS 6mm.

Table 1. Quantitative results from automated crystal assessment in patients with early-moderate BCD (Yuzawa stage 1 and 2). Crystal size and crystal distance from the fovea for all crystals within the 30-by-30 deg crop are reported in pixels as median and IQR. The raw number of crystals is reported for ETDRS circles 1, 3 and 6mm. Conversion from pixels to degrees is also reported.

ID	*Visit N*	*Age*	*Pixel to degrees*	*Crystal size (pixels)*	*Crystal distance (pixels)*	*ETDRS 1mm*	*ETDRS 3mm*	*ETDRS 6mm*
9	1	17	16.3	3 (2-5.8)	126 (69.3-179.8)	16	61	141
20	1	29	16.6	3 (1-5)	182 (108-224)	1	41	144
	2	30		4 (2-6)	144 (99-199)	9	120	423
	3	31		4 (2-7)	191 (141.8-229)	2	34	163
27	1	31	16.5	3 (2-6)	119 (78-224.5)	17	106	221
12	1	56	16.45	3 (1.5-7.5)	102 (84-137.3)	0	1	3
	2	58		4 (2.3-6)	111 (102.8-204)	0	4	23
	3	59		2 (1-4)	116.5 (104-251)	0	1	20

References

1. Kirkpatrick JNP, Spencer T, Manivannan A, Sharp PF, Forrester JV. Quantitative image analysis of macular drusen from fundus photographs and scanning laser ophthalmoscope images. *Eye.* 1995;9(1):48-55.
2. Mittal D, Kumari K. Automated detection and segmentation of drusen in retinal fundus images. *Computers & Electrical Engineering.* 2015;47:82-95.
3. Mora AD, Vieira PM, Manivannan A, Fonseca JM. Automated drusen detection in retinal images using analytical modelling algorithms. *BioMedical Engineering OnLine.* 2011;10(1):59.
4. Kato Y, Inoue M, Hirakata A. Quantitative comparisons of ultra-widefield images of model eye obtained with Optos(®) 200Tx and Optos(®) California. *BMC ophthalmology.* 2019;19(1):115-115.
5. Sagong M, van Hemert J, de Koo LCO, Barnett C, Sadda SR. Assessment of accuracy and precision of quantification of ultra-widefield images. *Ophthalmology.* 2015;122(4):864-866.
